# 601. Assessment of a Nursing and Pharmacy Collaborative Outpatient Parenteral Antimicrobial Therapy Management Program

**DOI:** 10.1093/ofid/ofab466.799

**Published:** 2021-12-04

**Authors:** Alice N Hemenway, Rebecca L Stewart

**Affiliations:** 1 University of Illinois Chicago College of Pharmacy- Rockford Health Sciences Campus, Rockford, IL; 2 Mercyhealth Javon Bea Hospital, Rockford, IL

## Abstract

**Background:**

At our facility a collaborative team of nurse and pharmacist manage patients receiving outpatient parenteral antimicrobial therapy (OPAT). This project aims to characterize this collaboration and assess the effectiveness by reviewing interventions made by the nurse and pharmacist, and assessing patient outcomes such as OPAT or infection related hospital admissions or ED visits, infection clearance, and mortality.

**Methods:**

A retrospective cohort study was performed on patients started on OPAT between 1/1/19 and 12/31/20. This time period was split into three: Period 1 where the clinic only included the PharmD and they saw patients for in-person appointments, Period 2 where the clinic included both the OPAT RN and PharmD and the PharmD performed in-person appointments, and Period 3 where the clinic included both but due to COVID the in-person PharmD appointments were on hold. OPAT or infection related hospital admissions, ED visits, infection clearance, and death were compared for each period.

**Results:**

A total of 388 patients were included in the review. There were 158 (40.7%) and 148 (38.1%) OPAT-related phone calls from the PharmD and RN, respectively. The two most common reasons for both PharmD and RN phone calls were a medication stop order/confirmation, and weekly lab obtainment. The third most common reason for the PharmD was dose change, and for the RN it was patient education. During Periods 1 and 2 the PharmD had in-person appointments with 28.9% of patients. The overall OPAT/infection related hospital admission and ED visit rates were 7.7% and 5.4%, respectively. Periods 2 and 3, which utilized the combined efforts of RN and PharmD, had consistently lower hospital admissions related to OPAT/infection (46-50% vs 62% Period 1), and ED visits due to OPAT/infection (33-36% vs 47% for Period 1). Clearance of infection was high for all 3 periods (89-95%), and mean mortality was low (2.1%).

**Conclusion:**

Collaborative management allowed for the nurse and pharmacist to function as substitutes for each other without losing the specific focus of their specialties, with the RN performing more patient education, and the PharmD performing more medication dosing. The collaboration had positive effects on OPAT patient outcomes.

**Disclosures:**

**All Authors**: No reported disclosures

